# Correlation between vascular endothelial growth factor pathway and immune microenvironment in head and neck squamous cell carcinoma

**DOI:** 10.1186/s12885-021-08547-4

**Published:** 2021-07-20

**Authors:** Chao Zhu, Liqun Gu, Zelong Liu, Jiang Li, Mianfeng Yao, Changyun Fang

**Affiliations:** 1 Department of Stomatology, Xiangya Hospital, Central South University, Changsha, China; 2 Department of Pediatric Stomatology, Xiangya Stomatological Hospital, Central South University, Changsha, China; 3 Division of Interventional Ultrasound, The First Affiliated Hospital of Sun Yat-sen University, Guangzhou, China

**Keywords:** Vascular endothelial growth factor, Immune microenvironment, Head and neck squamous cell carcinoma, Immunotherapy

## Abstract

**Background:**

Immunotherapy is a crucial therapeutic approach in oncology. However, most patients with head and neck squamous cell carcinoma (HNSCC) do not derive benefit from immunotherapy. Vascular endothelial growth factor (VEGF)/VEGF Receptor 2 (VEGFR2) signaling pathway is one of the most important pathways regulating angiogenesis in tumor. The combination of immunotherapy and anti-angiogenic therapy is considered to improve efficacy of immunotherapy. The correlation between VEGF signaling pathway and tumor immune microenvironment in HNSCC patients is unclear.

**Methods:**

We utilized RNA sequencing and clinical data of HNSCC patients from the TCGA database to study the correlation between VEGF signaling pathway and tumor immune microenvironment, on aspect of immune cell infiltration, immune-related gene expression profiling and immune-related biological pathways.

**Results:**

We observed that VEGF signaling pathway is positively correlated with immune cell infiltration, immune-related gene expression profiles, and the prognosis of HNSCC patients. The functional enrichment analysis of differentially expressed genes between different VEGF score subtypes detected multiple immune-related biological processes.

**Conclusion:**

Our findings suggested that combining anti-VEGF signaling pathway agents with immunotherapy, such as immune checkpoint inhibitors (ICI) therapy, may exhibit encouraging benefits in HNSCC.

**Supplementary Information:**

The online version contains supplementary material available at 10.1186/s12885-021-08547-4.

## Introduction

Head and neck cancer is the eighth most common cancer worldwide in 2020, which are largely head and neck squamous cell carcinoma (HNSCC) [1]. Human papillomavirus (HPV) has emerged as a novel risk factor for HNSCC. HPV-driven ones, especially oropharyngeal squamous cell carcinoma, feature distinct clinical and epidemiological characteristics compared with non-HPV induced HNSCC. HPV-positive HNSCC generally responds better to anti-tumor treatment. In contrast with HPV-negative HNSCC, HPV-positive HNSCC has a favorable prognosis [2].

Over the last decade, cancer immunotherapy, such as immune checkpoint inhibitors (ICI) therapy, has brought significant survival improvements [3]. Although only a subset of HNSCC patients responded to ICI therapy, the overall survival (OS) of responders was significantly improved [4, 5]. Based on these promising outcomes, the immune checkpoint inhibitors nivolumab and pembrolizumab have both been approved by U.S. Food and Drug Administration (FDA) for the treatment of recurrent or metastatic HNSCC. Recently, Pembrolizumab has been approved as first line treatment, based on a randomized, multicenter, open-label study, KEYNOTE-048 (NCT02358031) [6]. Despite the benefit of multimodal therapy, the prognosis of patients remains poor [7]. Thus, identification of predictive biomarkers and rational combination therapy are needed to improve outcomes.

Angiogenesis is crucial for tumor growth, proliferation, and metastasis. Vascular endothelial growth factor (VEGF) is the principle regulator of angiogenesis, activating pro-angiogenic signaling pathways and regulating new blood vessel formation by binding to its main receptor, VEGFR2 [8]. Targeting angiogenesis signaling pathways has been approved as standard therapy for multiple tumor types [9]. However, anti-angiogenic therapy failed to demonstrate significant anti-tumor activity or improved clinical efficacy in HNSCC. The interest in angiogenesis as a therapeutic target remains.

Accumulating evidence showed that VEGF not only promotes angiogenesis but also mediates immunosuppressive microenvironment [10]. This suggested that anti-VEGF therapy could stimulate the immune response and enhance the efficacy of immunotherapy. Preclinical studies showed that anti-VEGF treatment has the potential to reprogram the tumor immune microenvironment away from an immunosuppressive profile [11]. A phase I study combining ipilimumab and bevacizumab indicated that anti-VEGF could stimulate the immune system, and immunotherapy could inhibit angiogenesis [12]. It is apparent that combination of these two types of therapies could enhance both anti-tumor effects. Combination anti-angiogenic agents with immunotherapy has established benefit in multiple tumor types [13]. The advantage of this combination remains unclear in HNSCC.

Therefore, we investigated the correlation between VEGF signaling pathway and tumor immune microenvironment, and its association with survival of HNSCC patients using data obtained from The Cancer Genome Atlas (TCGA). The goal is to better understand the correlation between VEGF signaling pathway and tumor immune microenvironment.

## Material and methods

### Data sources

RNA sequencing and clinical data of 522 HNSCC patients were obtained from TCGA data portal (https://portal.gdc.cancer.gov) and eBioPortal (https://www.cbioportal.org/). 209 samples were excluded due to missing follow-up information and HPV status. Finally, 255 HPV-negative samples and 58 HPV-positive samples were included in this study.

### Molecular signatures and single-sample gene set enrichment analysis (ssGSEA) scores

The enrichment score of the VEGF signaling pathway and score of apoptosis were calculated using ssGSEA method. The gene set of *Homo sapiens* KEGG_VEGF_SIGNALING_PATHWAY and KEGG_APOPTOSIS was downloaded from the GSEA Molecular Signatures Database (MSigDB) v7.2 [14, 15]. Data on stromal fraction, and leukocyte fraction were obtained from a previously published study from the TCGA group [16]. We calculated expression profiles of 782 genes from 28 types of immune cell to quantify the infiltration of immune cells [17]. The degree of immune cell infiltration was estimated by the ssGSEA method through the Gene Set Variation Analysis (GSVA) package and visualized by heatmap package in R software v4.0.5 [18, 19]. The stromal, immune, and estimate scores were downloaded from ESTIMATE database [20]. Data of metastatic gene-set and proliferation score was obtained from two previous studies [16, 21].

### Immune-related gene expression profiling

We calculated the gene expressions of 75 immune markers related to the immune response in the tumor microenvironment [16].

### Differential gene expression analysis and gene ontology (GO) terms enrichment analysis

To identify differentially expressed genes (DEGs) between VEGF-high subtype and VEGF-low subtype, RNA sequencing data was performed using the limma package with cutoff of |log2FC| ≥ 1.0 and FDR < 0.05 [22]. Gene Ontology (GO) terms enrichment analysis of DEGs were analyzed using the Metascape [23] and visualized by ggplot2 package [24].

### Protein-protein interaction (PPI) network of immune-related DEGs

We extracted immune-related DEGs from the identified DEGs between VEGF-high subtype and VEGF-low subtype. Based on the immunologically relevant list of genes from the Immunology Database and Analysis Portal [25], we utilized the STRING database to construct PPI network among the immune-related DEGs [26], and rebuilt the PPI network by Cytoscape [27]. We applied MCODE algorithm to this network to identify neighborhoods where proteins are densely connected.

### Statistical analysis

Data comparison between the two VEGF pathway subtypes was performed via two-tailed t test and multiple t tests with FDR < 0.05 for continuous comparisons. The correlation between the VEGF pathway scores and the ssGSEA scores of 28 immune cells was determined by Pearson correlation test. The correlation between the VEGF pathway scores and immune signatures scores was visualized and calculated using the corrplot package [28]. Positive correlations were displayed in blue and negative correlations in red color. Heatmap was used to visualize and compare immune cell infiltration patterns and immune signatures across different VEGF signaling pathway subtypes. OS was plotted using Kaplan-Meier curves and calculated using the Multivariate Cox regression analysis. In all analyses, a *P* value of a two-tailed test less than 0.05 was thought to be statistically significant. All statistical analyses were conducted by GraphPad Prism v8.0.2 and R software v4.0.5.

## Results

### Correlation between immune cell infiltration and VEGF pathway score subtypes

**Fig. 1:**
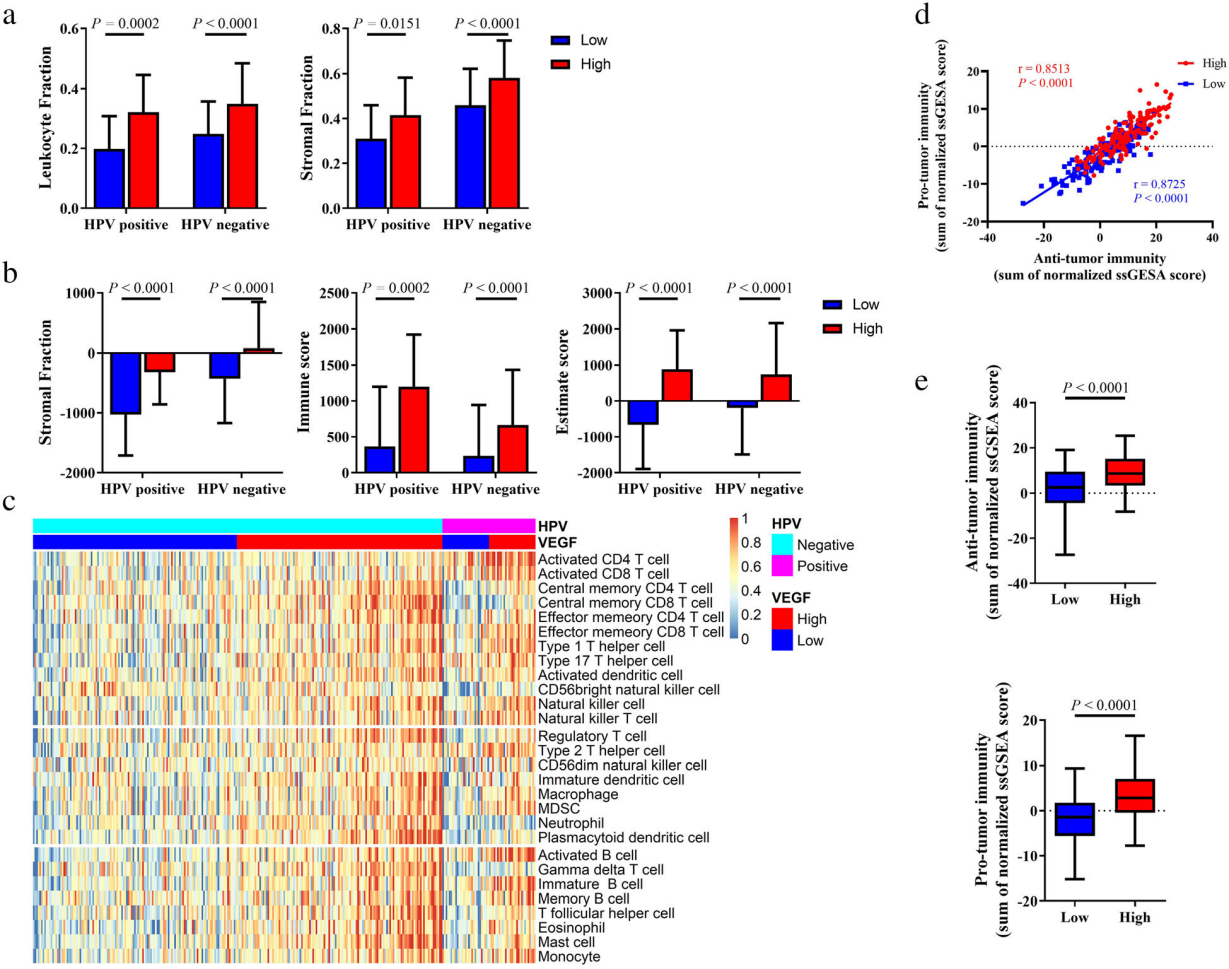
Immune cell infiltration patterns of two VEGF score subtypes in HPV-positive and -negative HNSCC. (**a**) Stroma fraction and leukocyte fraction of the two VEGF score subtypes. (**b**) Immune score, stromal score, and Estimate score of the two VEGF score subtypes. (**c**) Heatmap of immune cell infiltration, including both anti-tumor immune cells and pro-tumor immune cells. (**d**) A positive correlation between these two categories of immune cells in VEGF cluster. (**e**) Anti-tumor immunity and pro-tumor immunity of the two VEGF score subtypes. All *P* values for significance (< 0.05) represent comparisons via two-tailed t test and multiple t tests with FDR < 0.05 for continuous comparisons. All r values represent Pearson correlation coefficients

**Fig. 2:**
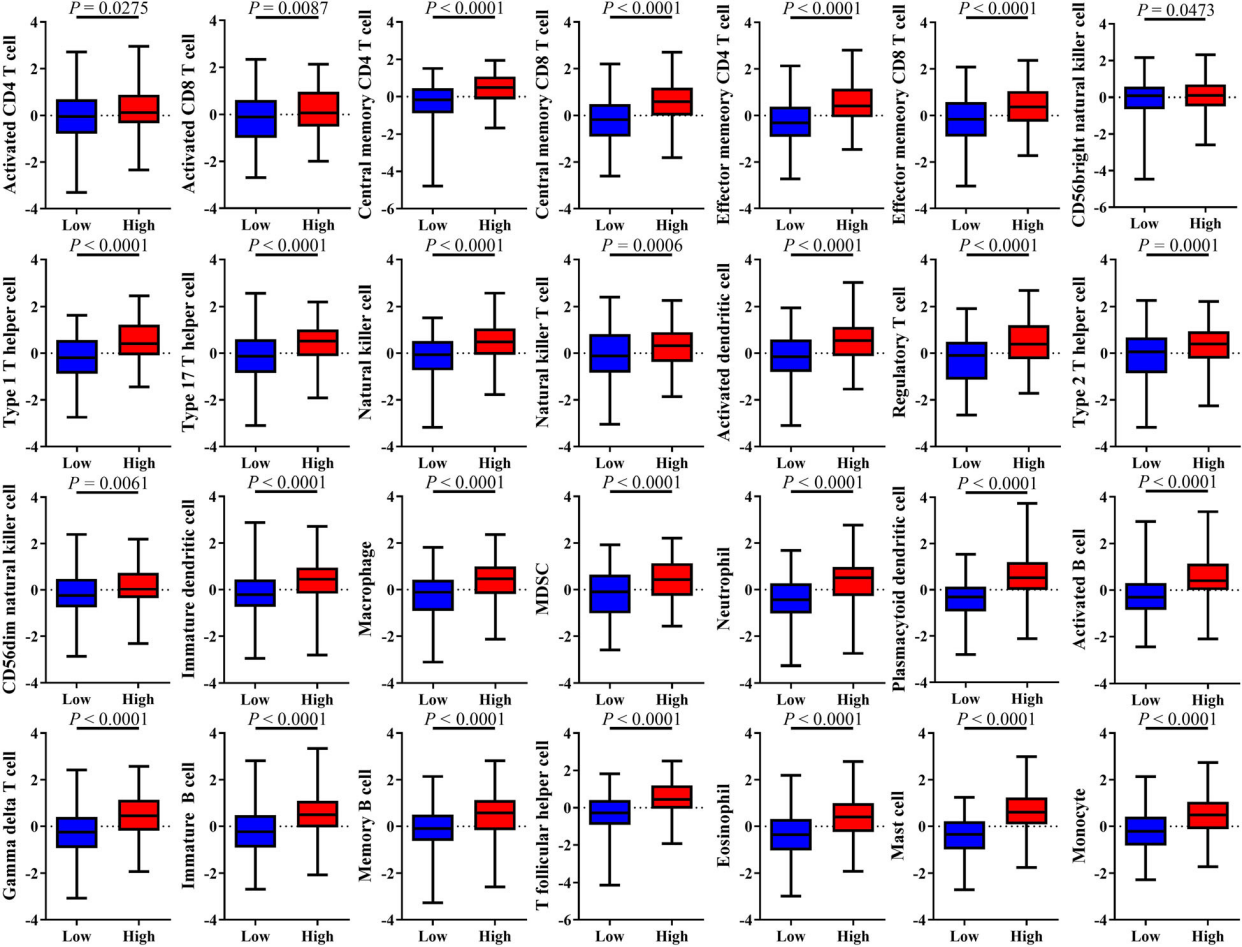
Infiltration of 28 immune cells in two VEGF score subtypes. All *P* values for significance (< 0.05) represent comparisons via two-tailed t test for continuous comparisons

We calculated and visualized the enrichment score of the VEGF signaling pathway (Additional file 1), and divided HNSCC patients into two VEGF pathway score subtypes with median cutoff: the VEGF-low score subtype with the bottom half score (*n* = 127 in HPV-negative HNSCC and *n* = 29 in HPV-positive HNSCC) and the VEGF-high score subtype with the top half score (*n* = 128 in HPV-negative HNSCC and *n* = 29 in HPV-positive HNSCC). We compared stromal fraction and leukocyte fraction between these two VEGF score subtypes. The results showed that higher stromal fraction, and higher leukocyte fraction in the VEGF-high score subtype in both HPV-positive and HPV-negative HNSCC (Fig. 1A, *P* = 0.0002, *P* < 0.0001; *P* = 0.0144, *P* < 0.0001, respectively). Using ESTIMATE database, we observed that higher immune score, stromal score, and estimate score in the VEGF-high score subtype in both HPV-positive and HPV-negative HNSCC (Fig. 1B). Subsequently, we calculated 28 types of immune cell infiltration using ssGSEA method. The VEGF-high score subtype showed relatively higher immune cell infiltration, including cells with anti-tumor activity and immunosuppressive activity regardless of HPV status (Fig. 1C). So, we performed Pearson correlation test in all patient samples, we found that a positive correlation between these two categories of immune cells in VEGF clusters (Fig. 1D, r = 0.8725 *P* < 0.0001; r = 0.8513, *P* < 0.0001, respectively). We compared these two categories of immune cells in different VEGF score subtypes and observed that the VEGF-high score subtype featured both higher anti-tumor immunity and pro-tumor immunity (Fig. 1E, *P* < 0.0001, *P* < 0.0001, respectively). 28 immune cells showed significant higher immune cell infiltration in the VEGF-high score subtype (Fig. 2). Positive correlation was found between VEGF pathway score and the ssGSEA score of 28 immune cells using Pearson correlation test (Additional file 2).

### Correlation between immune-related gene signatures and VEGF pathway score subtypes

**Fig. 3:**
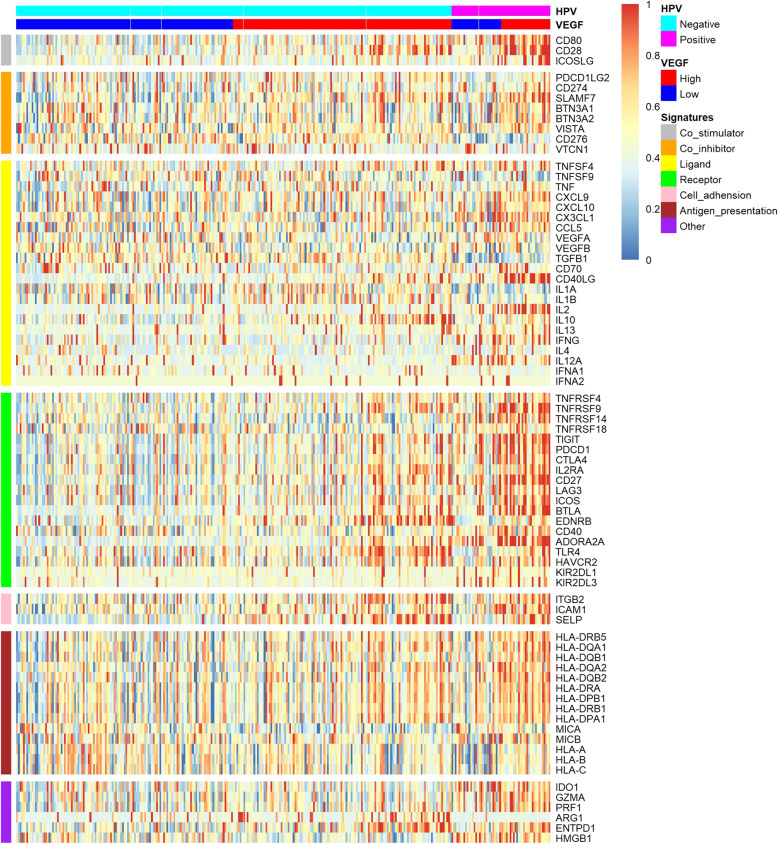
75 immune-related signatures expression profiling in VEGF pathway score subtypes

**Fig. 4:**
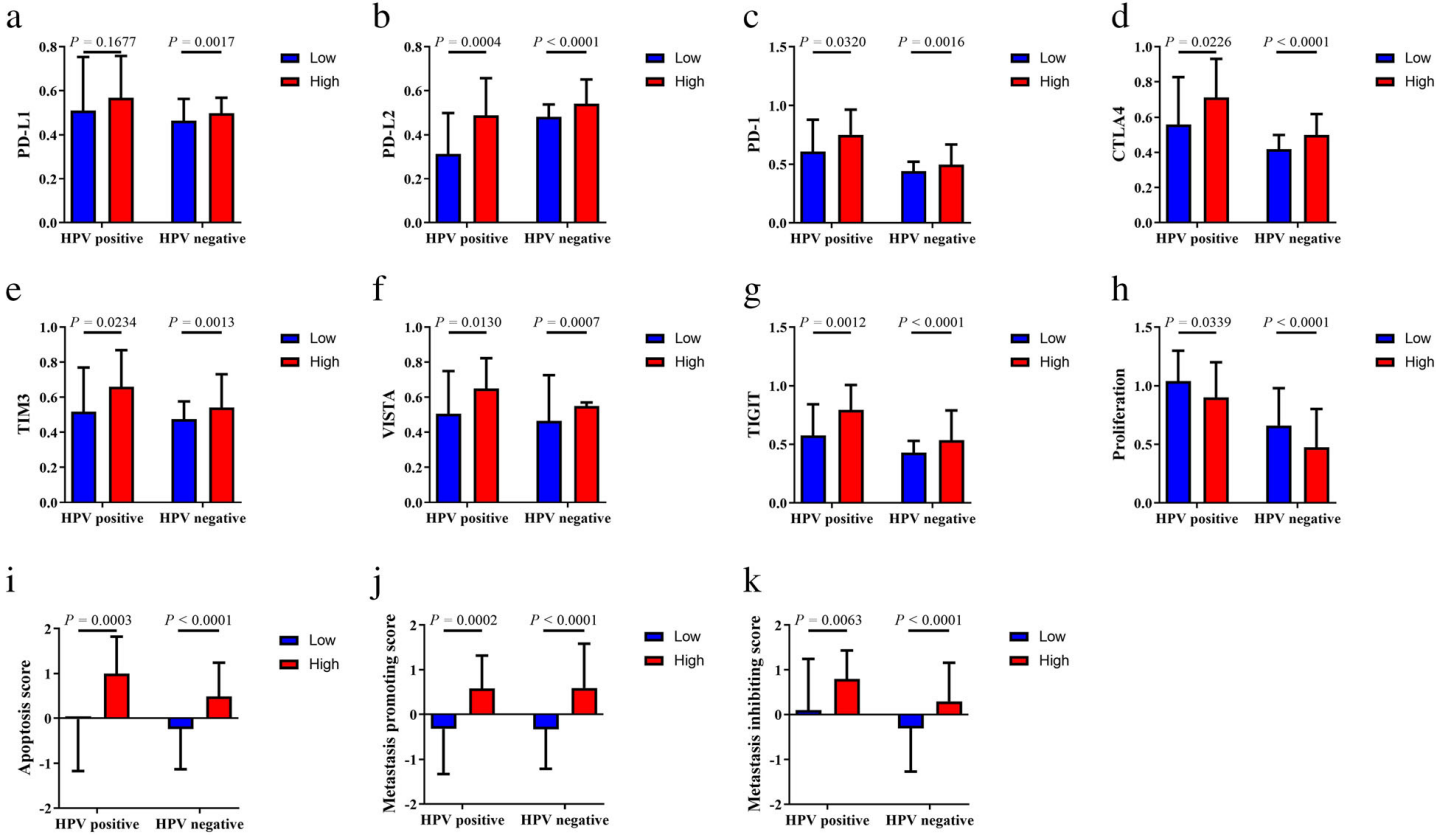
The expression level of immune checkpoint molecules and scores of proliferation, apoptosis, and metastasis in VEGF pathway score subtypes. All *P* values for significance (< 0.05) represent comparisons via multiple t tests with FDR < 0.05 for continuous comparisons

We evaluated expression profile of 75 immune-related genes in each VEGF pathway score subtype and the VEGF-high score subtype exhibited relatively higher expression of immune stimulatory and inhibitory signatures in both HPV-positive and HPV-negative samples (Fig. 3). We also found positive correlation between the expression of 75 immune-related genes and VEGF pathway scores in six groups: all HPV-positive patients, VEGF-high/HPV-positive subtype, VEGF-low/HPV-positive subtype, all HPV-negative patients, VEGF-high/HPV-negative subtype, VEGF-low/HPV-negative subtype (Additional file 3-8). When comparing the expression level of several important immune checkpoint molecules in VEGF cluster, we found that PD-L1 was expressed higher in VEGF-high score subtype in HPV-negative HNSCC, not in HPV-positive HNSCC (Fig. 4A, *P* = 0.0017; *P* = 0.1677, respectively). The expression level of PD-L2, PD-1, TIM3, VISTA, TIGIT was higher in VEGF-high score subtype independent of HPV status (Fig. 4B-G). We also found that lower proliferation score, higher apoptosis score, higher metastasis-promoting score, and higher metastasis-inhibiting score in the VEGF-high score subtype (Fig. 4H-K).

### Differentially expressed genes between VEGF score subtypes were enriched in immune-related GO terms

**Fig. 5:**
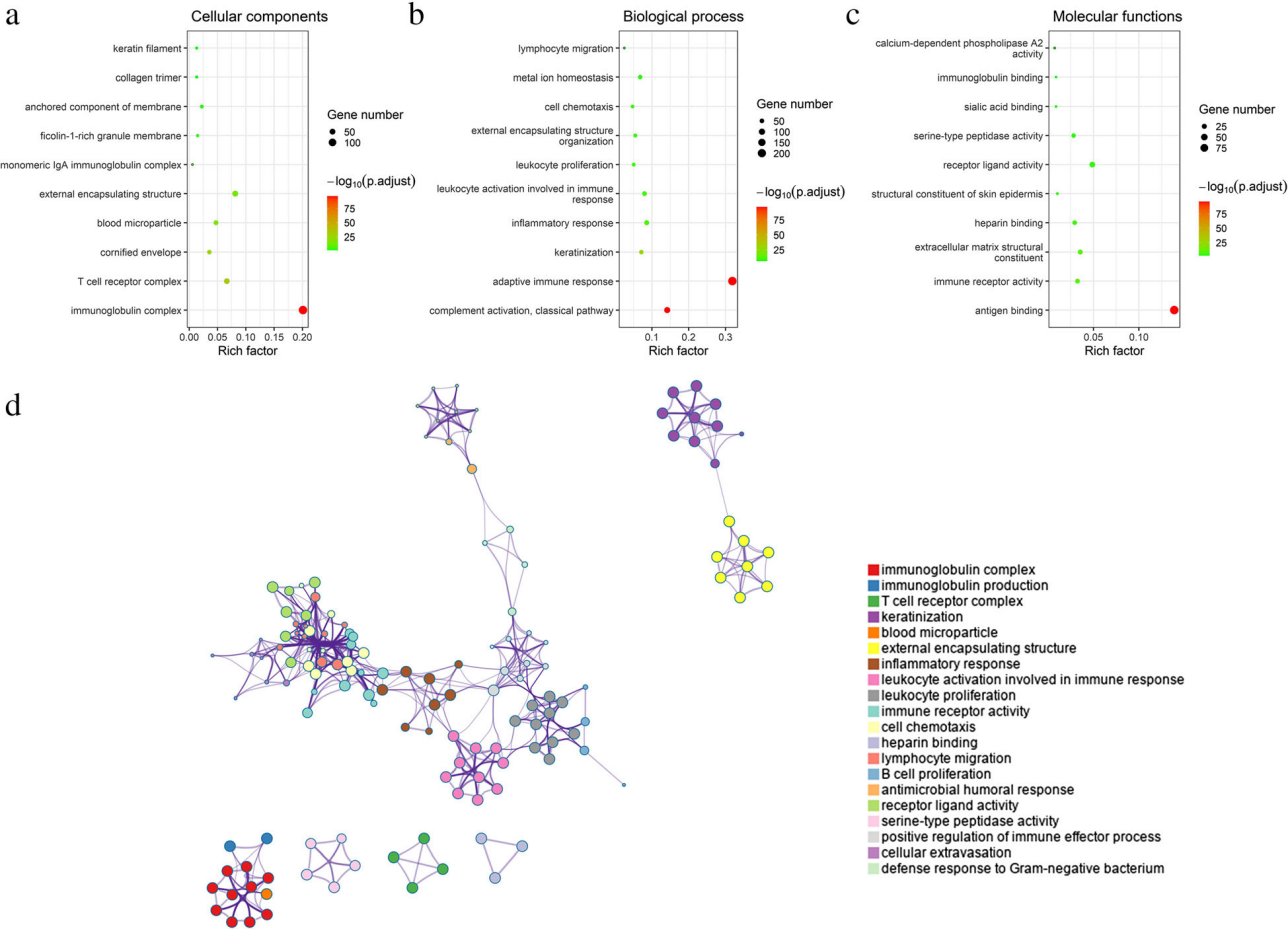
Functional enrichment analysis of DEGs between the two VEGF subtypes. (**a-c**) Functional enrichment analysis revealed that immune-related GO terms ranked top in cellular components, biological process, and biological process molecular functions. (**d**) The network of the top 20 GO summary terms

Functional enrichment analysis of DEGs in HPV-positive and HPV-negative samples were both enriched in immune-related GO terms (Additional files 9). Subsequently, we performed functional enrichment analysis of DEGs of all patients and revealed the following top immune related GO terms: immunoglobulin complex, T cell receptor complex and monomeric IgA immunoglobulin complex in cellular components (Fig. 5A); complement activation (classical pathway), adaptive immune response, and inflammatory response in biological process (Fig. 5B); antigen binding, immune receptor activity and receptor ligand activity in molecular functions (Fig. 5C). A network of immune-related GO terms was constructed based on the top 20 GO summary terms (Fig. 5D).

**Fig. 6:**
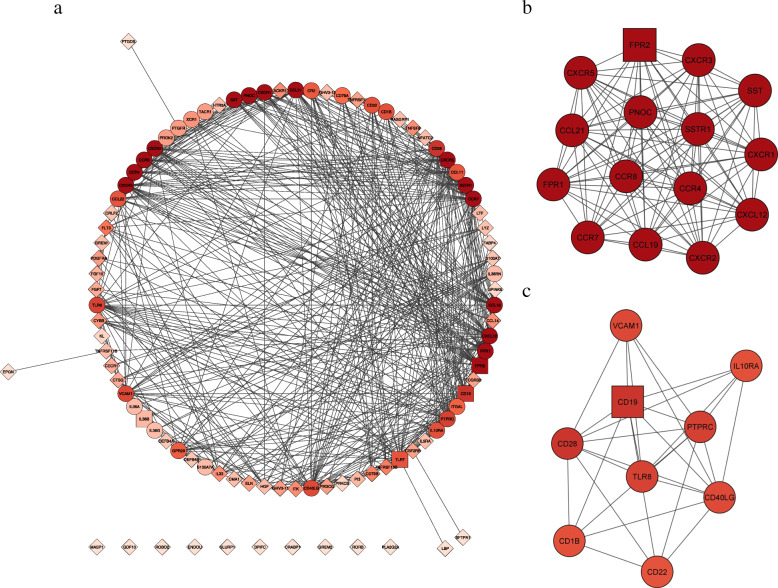
PPI network of immune-related DEGs. (**a**) PPI network consisted of 94 nodes and 501 edges, (**b**) cluster 1, MCODE score: 15, **c** cluster 2, MCODE score:7.25

We extracted 272 immune-related genes from the identified DEGs and constructed a PPI network which consists of 94 nodes and 501 edges (Fig. 6A). MCODE algorithm was applied to this network and two top clusters were found. Cluster 1 with MCODE score of 15 including 15 genes (CXCR1, CXCR2, CXCR3, CXCR5, PNOC, SST, SSTR1, FPR1, FPR2, CCR4, CCR7, CCR8, CCL19, CCL21, CXCL12) (Fig. 6B). Cluster 2 with MCODE score of 7.25 including 9 genes (CD18, CD19, CD22, CD28, CD40LG, PTPRC, TLR8, IL10RA, VCAM1) (Fig. 6C).

### Correlation of VEGF score subtypes, PD-1 and activated CD8^+^ T cells with survival of HNSCC patients

**Fig. 7:**
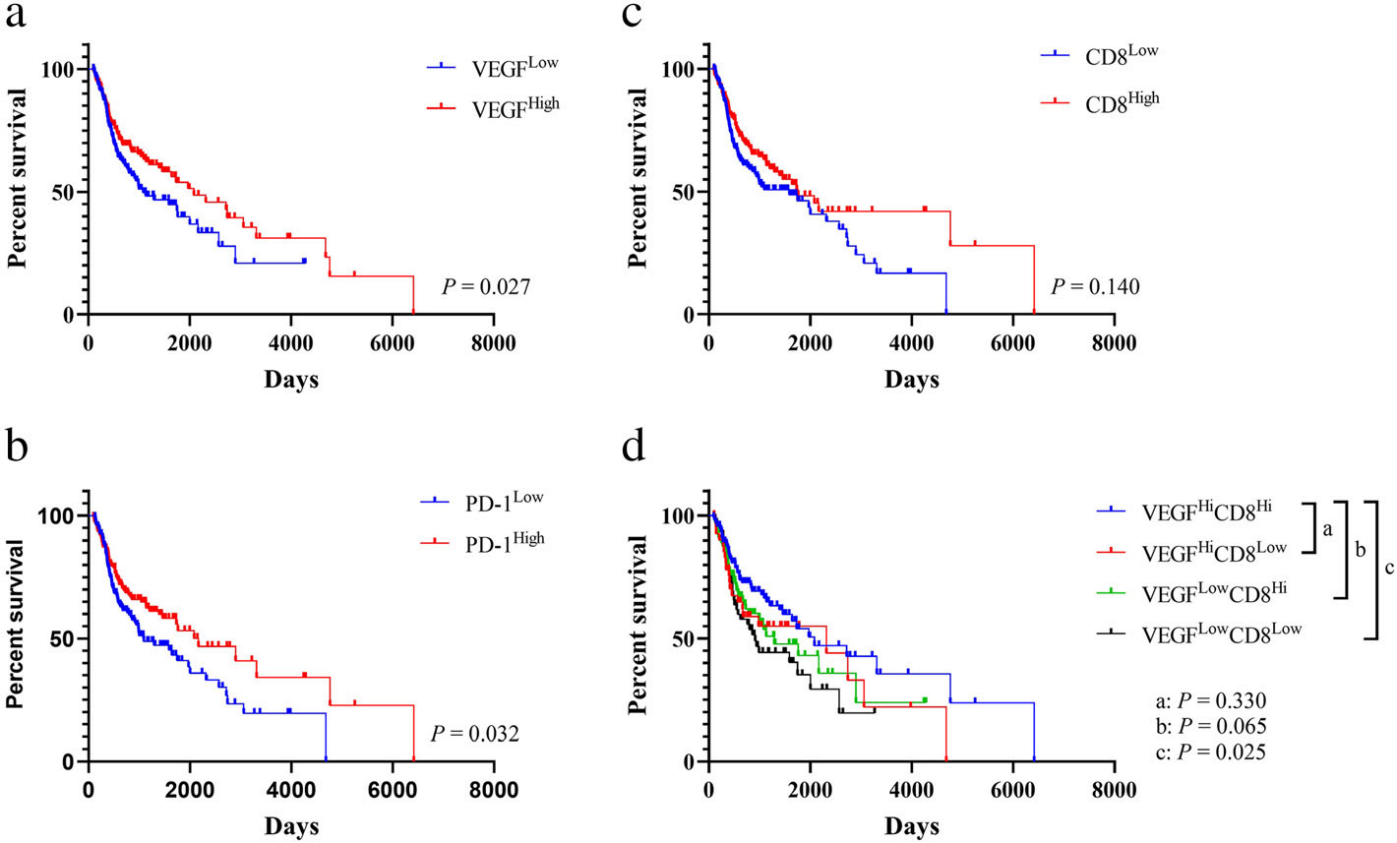
Survival analysis of HNSCC patients. (**a-c**) Correlation between VEGF pathway score, PD-1 expression, score of activated CD8+ T cells, and patient survival adjusted for HPV status. (**e**) Correlation between combined VEGF pathway score and score of activated CD8+ T cells, and patient survival adjusted for HPV status. *P* values for significance (< 0.05) calculated using multivariable Cox regression analysis

When compared to the VEGF-low score subtype, the VEGF-high score subtype showed a longer OS time (Fig. 7A, *P* = 0.027). Patients harboring higher level of PD-1 expression showed a significantly improved OS (Fig. 7B, *P* = 0.032), Based on the prognostic value of activated CD8^+^ T cells, we calculated ssGSEA score of activated CD8^+^ T cells in HNSCC patient samples and divided them into high activated CD8^+^ T cells group and low activated CD8^+^ T cells group with the median cutoff. Patients with high activated CD8^+^ T cells infiltration showed a trend towards better OS (Fig. 7C, *P* = 0.140). We then categorized HNSCC patients into four groups: VEGF^High^CD8^High^, VEGF^High^CD8^Low^, VEGF^Low^CD8^High^ and VEGF^Low^CD8^Low^. A better prognosis was observed in VEGF^High^CD8^High^ compared to VEGF^Low^CD8^Low^ and a borderline significant was found in VEGF^High^CD8^High^ compared to VEGF^Low^CD8^High^ (Fig. 7D, *P *= 0.025, *P *= 0.065, respectively).

## Discussion

Angiogenesis is considered as a crucial process in tumor development. Among the factors inducing tumor angiogenesis, the VEGF/VEGFR2 signaling pathway is one of the most important pathways. Because of a cascade of different signaling pathways involve in angiogenesis, so we considered the whole VEGF/VEGFR2 signaling pathway to represent angiogenesis status in tumor.

We profiled the immune cell infiltration and immune-related gene signatures patterns in HPV-positive and HPV-negative HNSCC tumors. We found that the VEGF-high score subtype infiltrated higher levels of immunosuppressive cells, such as Treg cell, macrophage, MDSC and immature dendritic cell. Interestingly, we also found higher anti-tumor immune cells in the VEGF-high score subtype, including activated CD4^+^ T cell, activated CD8^+^ T cell and nature killer cell. These findings indicated that both anti-tumor immune cells and immunosuppressive cells are infiltrated in the tumor microenvironment when VEGF signaling pathway related gene expressions are increased independent of HPV status. Together, these findings suggested that the VEGF-high score subtype is more immune inflamed, which might be likely to achieve benefits from immunotherapy.

We also observed that immune stimulatory factors and immune inhibitory factors were both higher expressed in the VEGF-high score subtype, which is consistent with immune cell infiltration phenotype. Among these immune signatures, the expressions of immune checkpoint genes, including PD-1, PD-L1, PD-L2, CTLA-4, TIM3, TIGIT and VISTA, were higher expressed in the VEGF-high score subtype. CTLA-4 is highly expressed on Treg cell, and CTLA-4 inhibitors have been shown to promote antitumor immunity [29]. TIGIT and TIM-3 have been linked to inhibit nature killer cell function. These indicated that VEGF-high score subtype might be likely to achieve the benefits from immune checkpoint inhibitors. Beyond PD-1/PD-L1 axis, targeting Treg cell and nature killer cell may exhibit beneficial activity in VEGF-high score subtype. In addition, anti-VEGF could normalize tumor vessels and improve the treatment efficacy of ICI therapy [11]. These indicated a potential rational of combining anti-VEGF therapy with ICI therapy.

We found that high VEGF score subtype shows higher immune cell infiltration, so-called hot tumor, which is correlated with OS. Similar findings had been observed by Hanna et al., who identified an inflamed subgroup of tumors with improved survival [30]. Another group found that tumor-infiltrating lymphocytes density and localization could predict the outcomes [31]. CD8^+^ T cells and nature killer cells, which were higher expressed in VEGF-high score subtype, correlated with better survival in HNSCC [32]. They usually indicated an activated phenotype despite the presence of multiple immunosuppressive cells and immune inhibitory factors. Accumulating data suggested a negative prognostic significance of PD-1 expression. By contrast, we found that PD-1 expression was associated with better survival outcomes, in agreement with other studies on HNSCCs [33, 34]. In addition, we found lower proliferation score, higher apoptosis score, higher metastasis-inhibiting score, and higher metastasis-promoting score in VEGF-high score subtype. Coutinho-Camillo et al. defined a pro-apoptotic cluster and an anti-apoptotic cluster, indicating a correlation between apoptosis and tumor behavior [35]. Genes in metastasis-promoting signature were mostly related to the function of Neutrophil cell, Treg cell, and macrophage; genes in metastasis-inhibiting signature were mostly related to interferon regulatory family and the function of T cell, nature killer cell. These could be the results of higher immune cell infiltration in VEGF-high score subtype.

The molecular mechanisms of VEGF pathway regulating immune response involves multiple signaling pathways. Functional enrichment analysis of DEGs revealed that immune-related GO terms were ranked top in biological process, cellular components, and molecular functions. The enrichment network of the top 20 summary GO terms revealed therapies with the potential to target immune-related biological pathways. Another PPI network showed that the immune-related DEGs enrich in cytokine and chemokine activities and immune cell - endothelial cell adhesion. This network revealed two important modules included 26 genes, further investigation of their role in head and neck cancer may prove beneficial. These included targeting CXCL12, CXCR1/CXCR2, and TLR8, which had shown therapeutic promise [36–38].

Combination of immunotherapy and anti-angiogenic therapy has established benefit in multiple tumor types [13]. Single anti-angiogenic therapy failed to demonstrate significant antitumor activity or improved clinical efficacy in HNSCC. The interest in combing anti-angiogenic therapy and immunotherapy remains. A phase IB/II trial of lenvatinib plus pembrolizumab in patients with multiple solid tumor types, including HNSCC, indicated promising preliminary efficiency with expected toxicities [39]. Several ongoing clinical trials are evaluating the benefits of combing these two types of therapies, including ongoing phase II trials of ramucirumab plus pembrolizumab and bevacizumab plus atezolizumab in recurrent/metastatic HNSCC (NCT03650764, NCT03818061).

One of the biggest limitations of our study is that the lack of information of histological data about the location of infiltrated immune cells. The concept of tumor-immune phenotype: immune-inflamed, immune-excluded, and immune-desert phenotypes, which is now widely accepted in solid tumor. Studies had revealed that immune-inflamed phenotype had a favorable prognosis [30]. Also, our findings are based on bioinformatics analysis, further experiments are needed to validate these findings.

## Conclusion

In summary, we investigated the correlation between VEGF signaling pathway and tumor immune microenvironment in HNSCC patients. Our findings revealed that combining anti-VEGF signaling pathway agents with immunotherapy, such as immune checkpoint inhibitors, may exhibit promising benefits in HNSCC.

## Supplementary Information

**Additional file 1.** Heatmap of VEGF/VEGFR2 signaling pathway.

**Additional file 2. **Correlation between the VEGF pathway scores and the ssGSEA scores of 28 immune cells. All r values represent Pearson correlation coefficients. Two-tailed *P* values are presented for significance (< 0.05).

**Additional file 3.** Correlation between the 75 immune-related signatures and VEGF pathway scores in HPV-positive patients. Positive correlations were displayed in blue and negative correlations in red color.

**Additional file 4.** Correlation between the 75 immune-related signatures and the high VEGF score subtype in HPV-positive patients. Positive correlations were displayed in blue and negative correlations in red color.

**Additional file 5.** Correlation between the 75 immune-related signatures and the low VEGF score subtypes in HPV-positive patients. Positive correlations were displayed in blue and negative correlations in red color

**Additional file 6.** Correlation between the 75 immune-related signatures and VEGF pathway scores in HPV-negative patients. Positive correlations were displayed in blue and negative correlations in red color.

**Additional file 7.** Correlation between the 75 immune-related signatures and the high VEGF score subtype in HPV-negative patients. Positive correlations were displayed in blue and negative correlations in red color.

**Additional file 8.** Correlation between the 75 immune-related signatures and the low VEGF score subtypes in HPV-negative patients. Positive correlations were displayed in blue and negative correlations in red color.

**Additional file 9. **Functional enrichment analysis of DEGs between two VEGF subtypes in HPV-positive and -negative HNSCC. (**A-C)** Functional enrichment analysis revealed that immune-related GO terms ranked top in cellular components, biological process, and biological process molecular functions in HPV-negative HNSCC. (**D-F**) Functional enrichment analysis revealed that immune-related GO terms ranked top in cellular components, biological process, and biological process molecular functions in HPV-positive HNSCC.

**Additional file 10.** Genset of KEGG_APOPTOSIS.

## Data Availability

All data used in this manuscript were from public data repositories. TCGA data portal (https://portal.gdc.cancer.gov/) and eBioPortal (https://www.cbioportal.org/). GSEA Molecular Signatures Database (https://www.gsea-msigdb.org/gsea/index.jsp). STRING database (https://string-db.org). ESTIMATE algorithm, (https://bioinformatics.mdanderson.org/estimate/index.html). Immunology Database and Analysis Portal (ImmPort) (https://immport.niaid.nih.gov).

## Abbreviations

HNSCC: Head and neck squamous cell carcinoma
VEGF: Vascular endothelial growth factor
VEGFR2: Vascular endothelial growth factor Receptor 2
HPV: Human papillomavirus
ICI: Immune checkpoint inhibitor
OS: Overall survival
TCGA: The Cancer Genome Atlas
ssGSEA: single-sample Gene Set Enrichment Analysis
GO: Gene Ontology
DEG: Differentially Expressed Gene
PPI: Protein-protein Interaction
