# Supplementary figures and images for: Correlation between vascular endothelial growth factor pathway and immune microenvironment in head and neck squamous cell carcinoma
Source: BMC Cancer. 2021 Jul 20;21:836. doi: 10.1186/s12885-021-08547-4 (PMC8290614; doi:10.1186/s12885-021-08547-4)

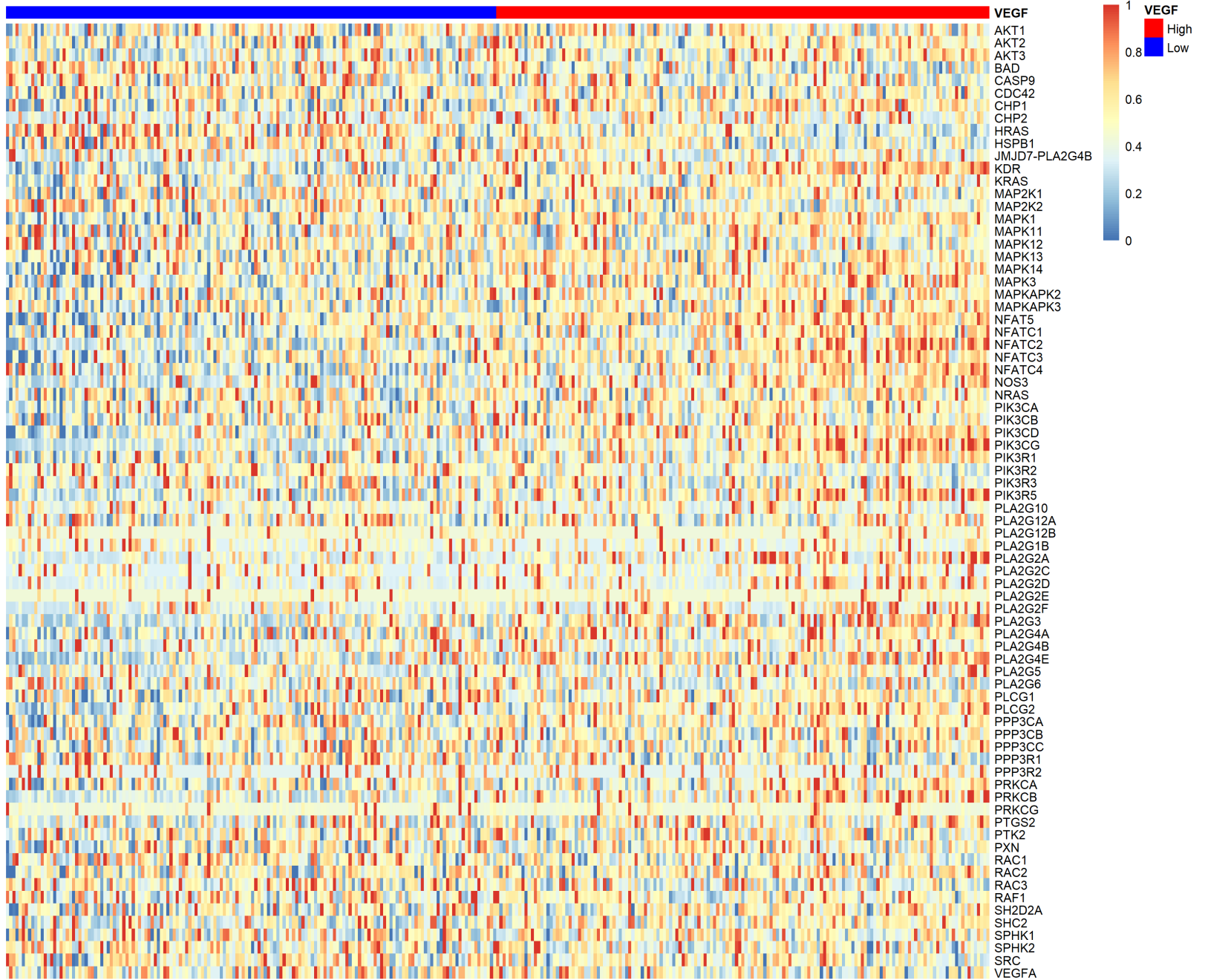

Supplement: Supplementary file 1 — Additional file 1. Heatmap of VEGF/VEGFR2 signaling pathway. [file 12885_2021_8547_MOESM1_ESM.pdf]

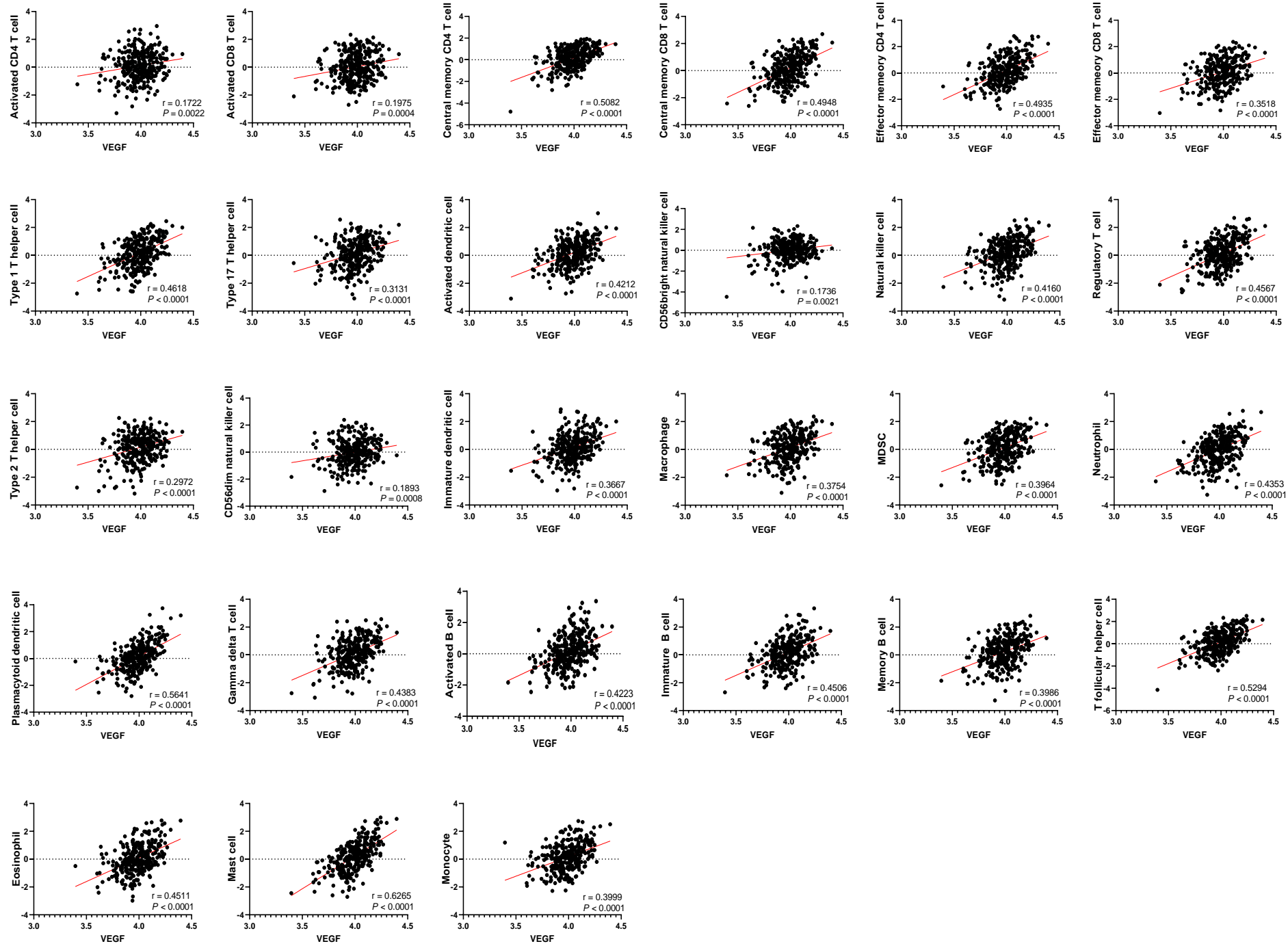

Supplement: Supplementary file 2 — Additional file 2. Correlation between the VEGF pathway scores and the ssGSEA scores of 28 immune cells. All r values represent Pearson correlation coefficients. Two-tailed P values are presented for significance (< 0.05). [file 12885_2021_8547_MOESM2_ESM.pdf]

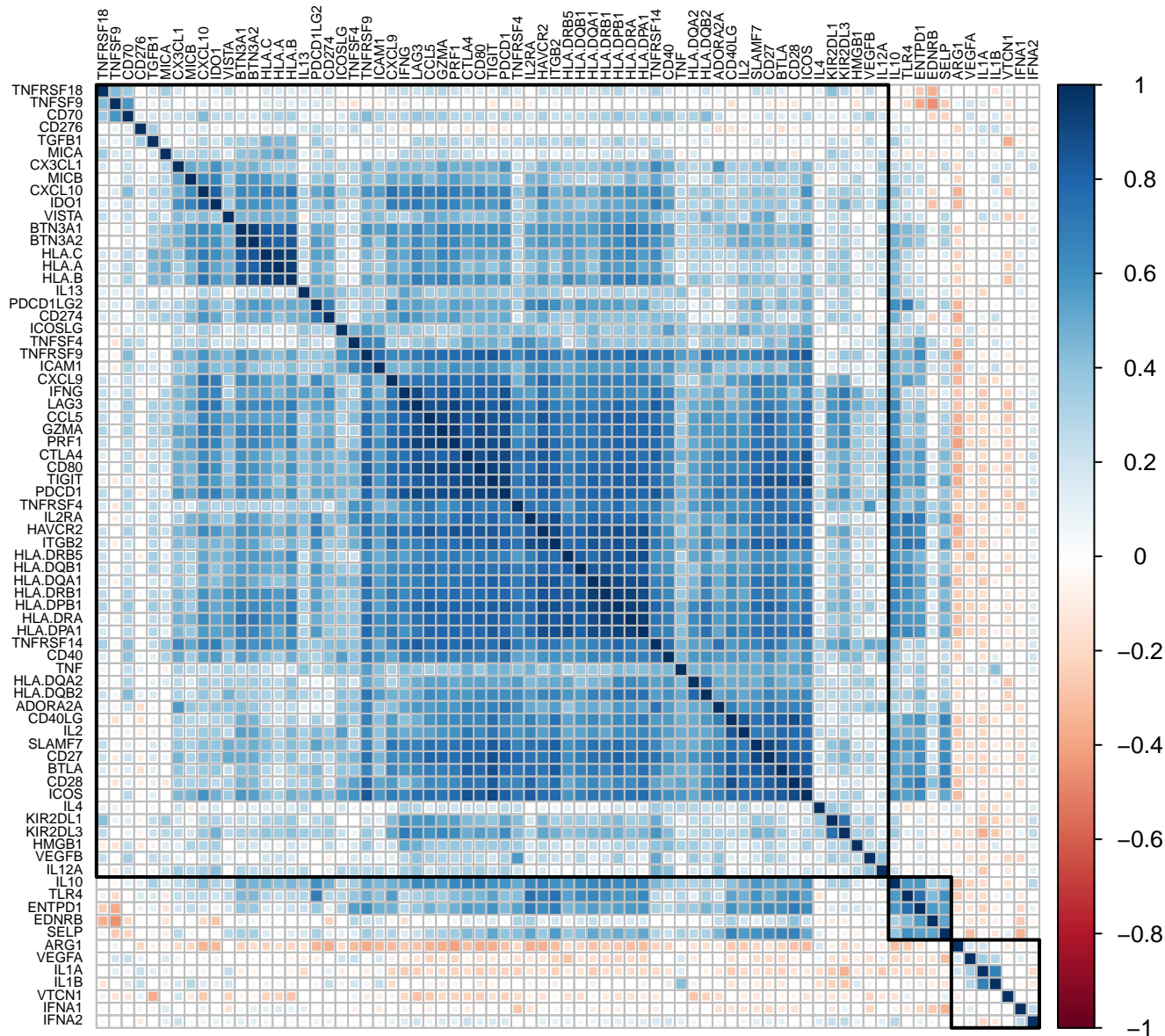

Supplement: Supplementary file 3 — Additional file 3. Correlation between the 75 immune-related signatures and VEGF pathway scores in HPV-positive patients. Positive correlations were displayed in blue and negative correlations in red color. [file 12885_2021_8547_MOESM3_ESM.pdf]

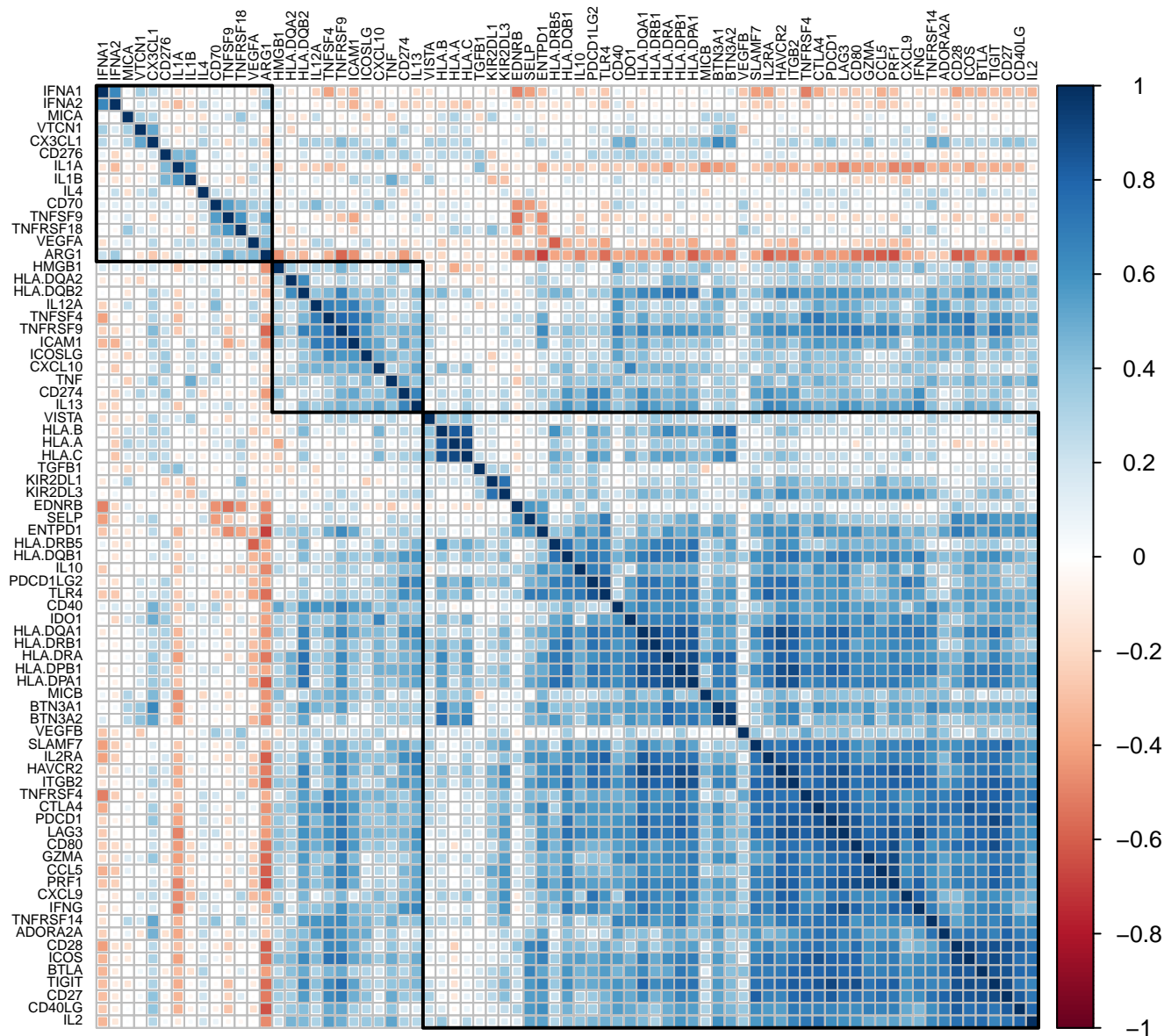

Supplement: Supplementary file 4 — Additional file 4. Correlation between the 75 immune-related signatures and the high VEGF score subtype in HPV-positive patients. Positive correlations were displayed in blue and negative correlations in red color. [file 12885_2021_8547_MOESM4_ESM.pdf]

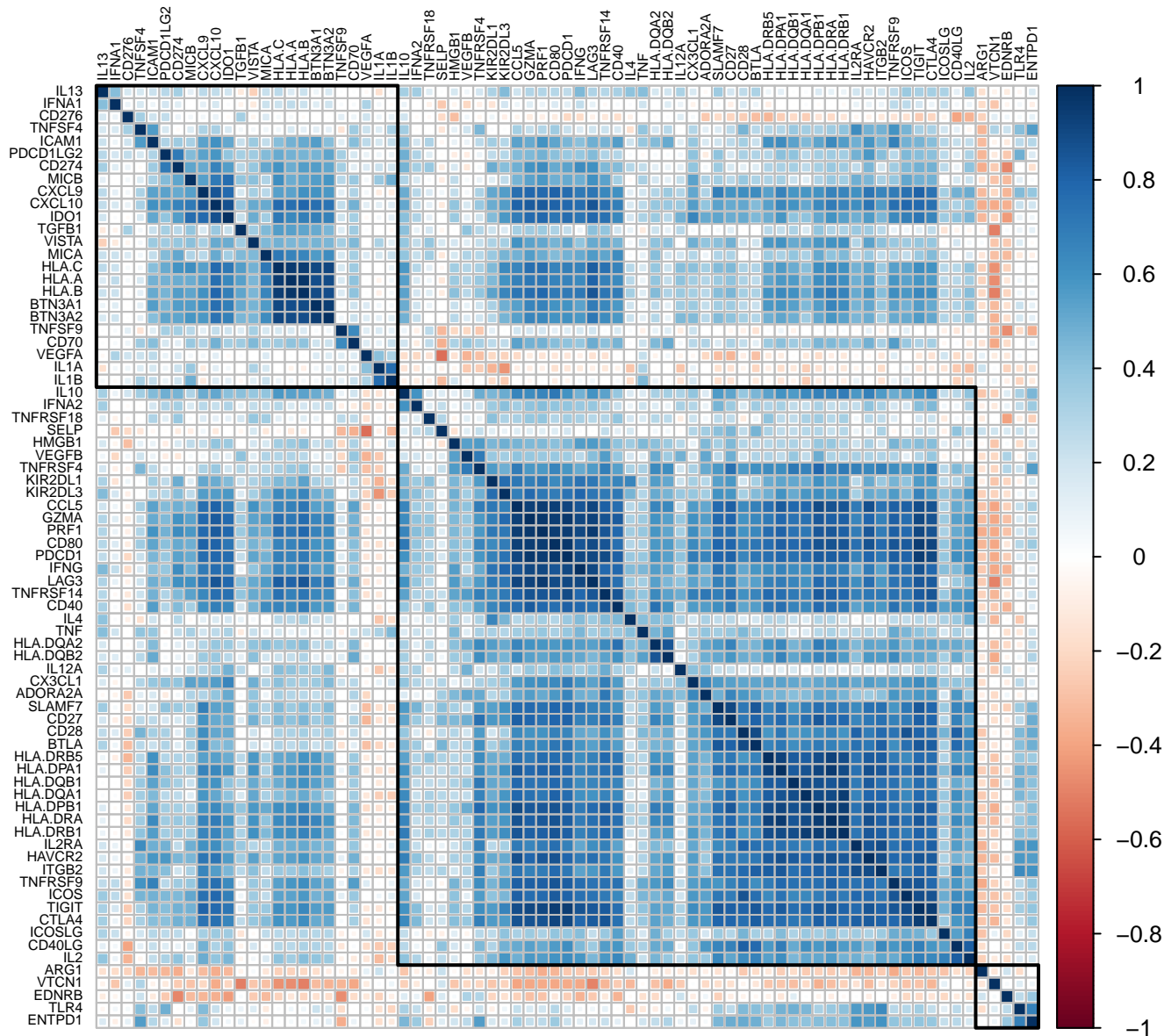

Supplement: Supplementary file 5 — Additional file 5. Correlation between the 75 immune-related signatures and the low VEGF score subtypes in HPV-positive patients. Positive correlations were displayed in blue and negative correlations in red color [file 12885_2021_8547_MOESM5_ESM.pdf]

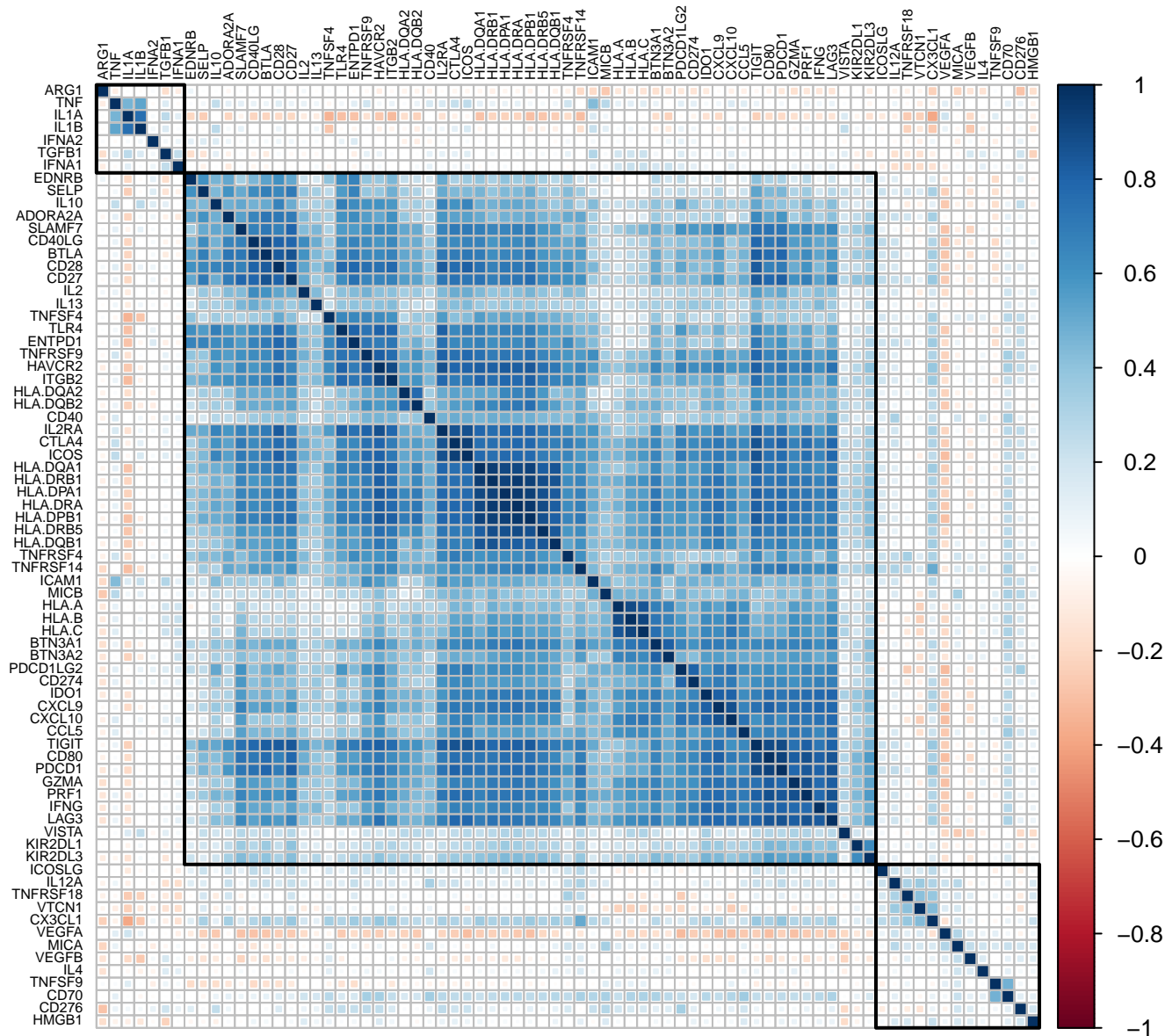

Supplement: Supplementary file 6 — Additional file 6. Correlation between the 75 immune-related signatures and VEGF pathway scores in HPV-negative patients. Positive correlations were displayed in blue and negative correlations in red color. [file 12885_2021_8547_MOESM6_ESM.pdf]

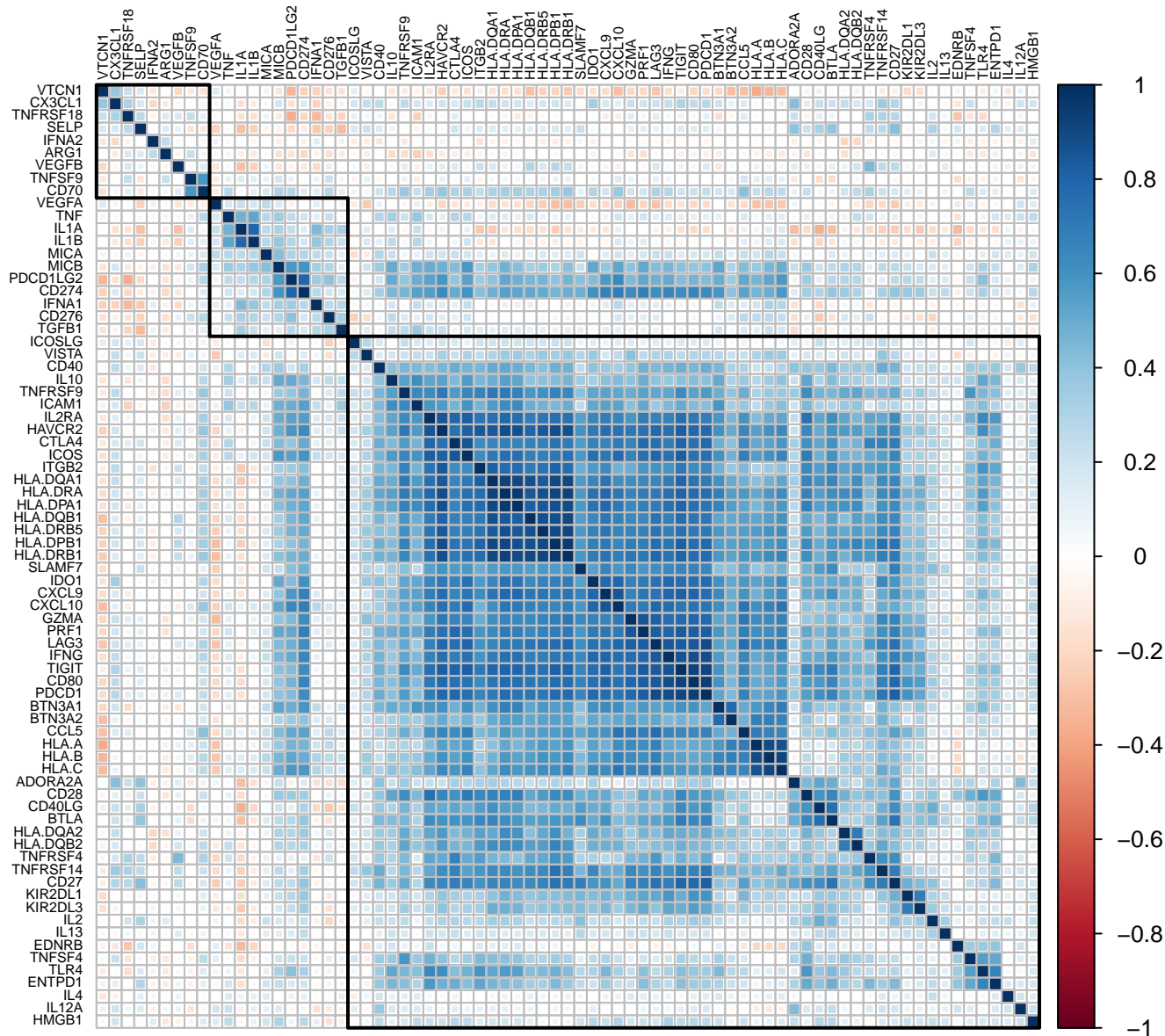

Supplement: Supplementary file 7 — Additional file 7. Correlation between the 75 immune-related signatures and the high VEGF score subtype in HPV-negative patients. Positive correlations were displayed in blue and negative correlations in red color. [file 12885_2021_8547_MOESM7_ESM.pdf]

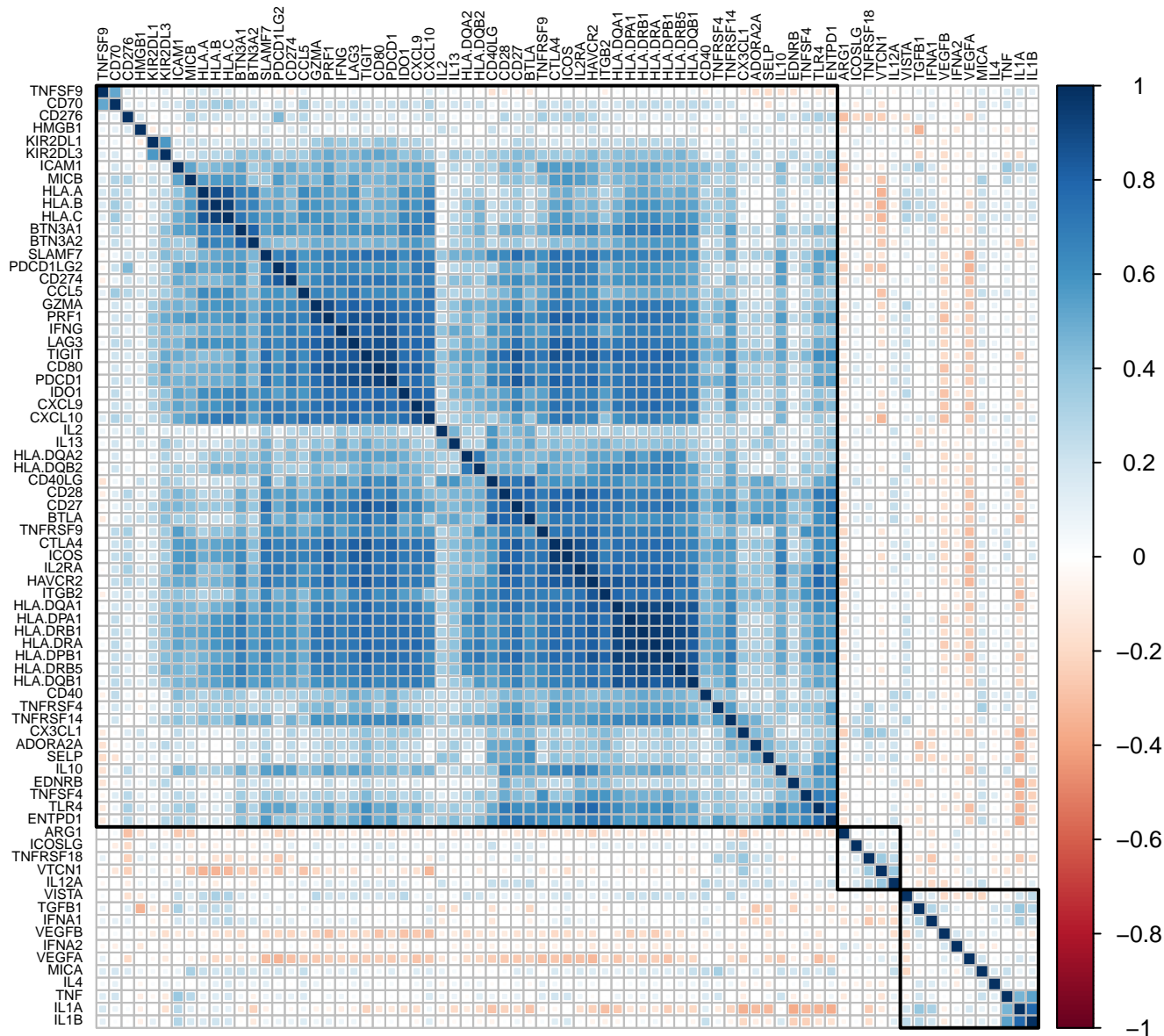

Supplement: Supplementary file 8 — Additional file 8. Correlation between the 75 immune-related signatures and the low VEGF score subtypes in HPV-negative patients. Positive correlations were displayed in blue and negative correlations in red color. [file 12885_2021_8547_MOESM8_ESM.pdf]
