# Supplementary material for: Correlation between vascular endothelial growth factor pathway and immune microenvironment in head and neck squamous cell carcinoma
Source: BMC Cancer. 2021 Jul 20;21:836. doi: 10.1186/s12885-021-08547-4 (PMC8290614; doi:10.1186/s12885-021-08547-4)
Supplement: Supplementary file 9 — Additional file 9. Functional enrichment analysis of DEGs between two VEGF subtypes in HPV-positive and -negative HNSCC. (A-C) Functional enrichment analysis revealed that immune-related GO terms ranked top in cellular components, biological process, and biological process molecular functions in HPV-negative HNSCC. (D-F) Functional enrichment analysis revealed that immune-related GO terms ranked top in cellular components, biological process, and biological process molecular functions in HPV-positive HNSCC. [file 12885_2021_8547_MOESM9_ESM.pdf]

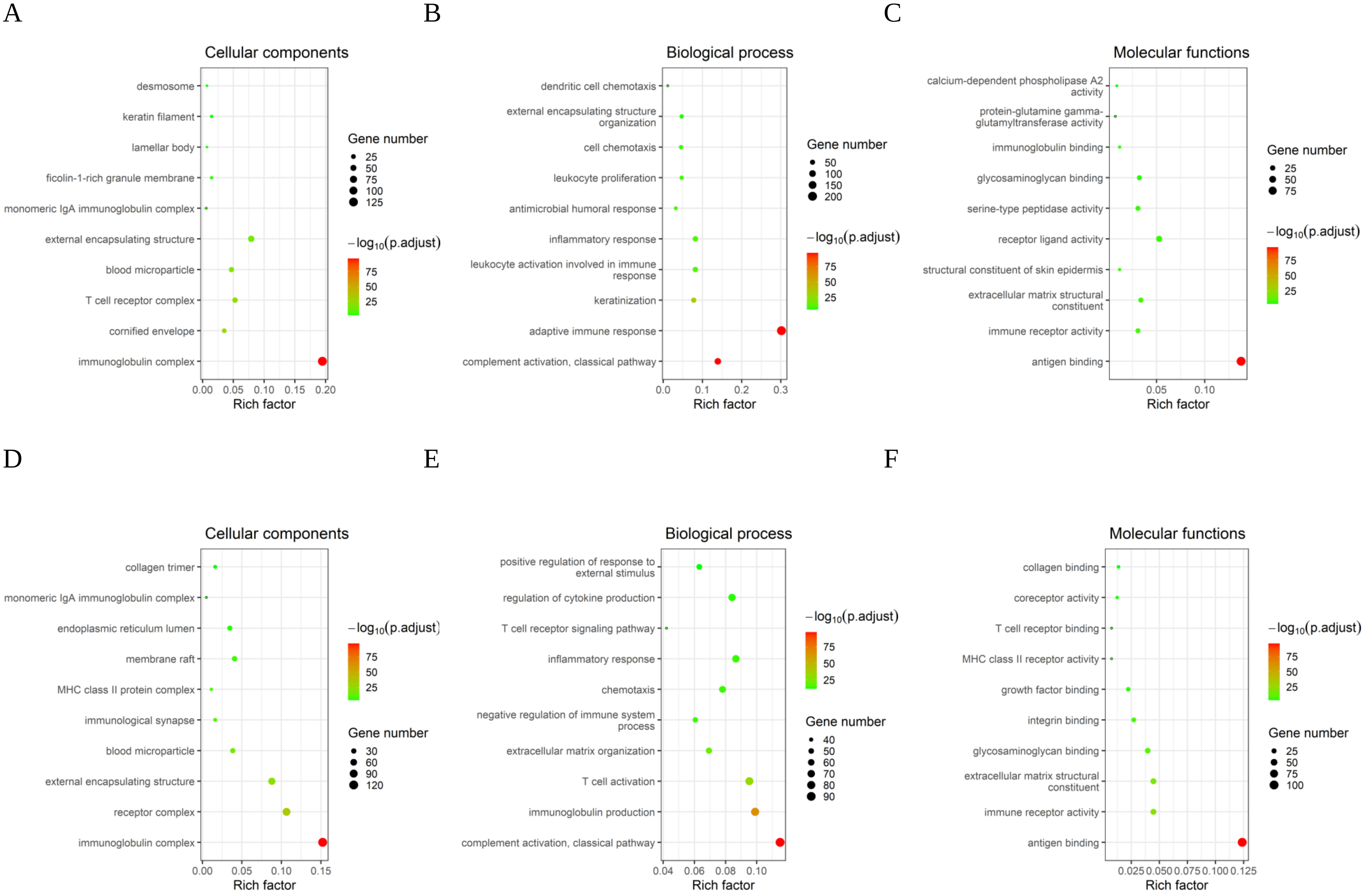
A
Cellular components
desmosome
keratin filament
lamellar body
ficolin-1-rich granule membrane
monomeric IgA immunoglobulin complex
external encapsulating structure
blood microparticle
T cell receptor complex
cornified envelope
immunoglobulin complex
0.00 0.05 0.10 0.15 0.20
Rich factor
Gene number
25
50
75
100
125
$-\log_{10}$(p.adjust)
75
50
25
B
Biological process
dendritic cell chemotaxis
external encapsulating structure organization
cell chemotaxis
leukocyte proliferation
antimicrobial humoral response
inflammatory response
leukocyte activation involved in immune response
keratinization
adaptive immune response
complement activation, classical pathway
0.0 0.1 0.2 0.3
Rich factor
Gene number
50
100
150
200
$-\log_{10}$(p.adjust)
75
50
25
C
Molecular functions
calcium-dependent phospholipase A2 activity
protein-glutamine gamma-glutamyltransferase activity
immunoglobulin binding
glycosaminoglycan binding
serine-type peptidase activity
receptor ligand activity
structural constituent of skin epidermis
extracellular matrix structural constituent
immune receptor activity
antigen binding
0.05 0.10
Rich factor
Gene number
25
50
75
$-\log_{10}$(p.adjust)
75
50
25
D
Cellular components
collagen trimer
monomeric IgA immunoglobulin complex
endoplasmic reticulum lumen
membrane raft
MHC class II protein complex
immunological synapse
blood microparticle
external encapsulating structure
receptor complex
immunoglobulin complex
0.00 0.05 0.10 0.15
Rich factor
$-\log_{10}$(p.adjust)
75
50
25
Gene number
30
60
90
120
E
Biological process
positive regulation of response to external stimulus
regulation of cytokine production
T cell receptor signaling pathway
inflammatory response
chemotaxis
negative regulation of immune system process
extracellular matrix organization
T cell activation
immunoglobulin production
complement activation, classical pathway
0.04 0.06 0.08 0.10
Rich factor
$-\log_{10}$(p.adjust)
75
50
25
Gene number
40
50
60
70
80
90
F
Molecular functions
collagen binding
coreceptor activity
T cell receptor binding
MHC class II receptor activity
growth factor binding
integrin binding
glycosaminoglycan binding
extracellular matrix structural constituent
immune receptor activity
antigen binding
0.025 0.050 0.075 0.100 0.125
Rich factor
$-\log_{10}$(p.adjust)
75
50
25
Gene number
25
50
75
100
